# Effect of Oral Docosahexaenoic Acid (DHA) Supplementation on DHA Levels and Omega-3 Index in Red Blood Cell Membranes of Breast Cancer Patients

**DOI:** 10.3389/fphys.2017.00549

**Published:** 2017-07-28

**Authors:** Alessio Molfino, Maria I. Amabile, Sara Mazzucco, Gianni Biolo, Alessio Farcomeni, Cesarina Ramaccini, Simonetta Antonaroli, Massimo Monti, Maurizio Muscaritoli

**Affiliations:** ^1^Department of Clinical Medicine, Sapienza University of Rome Rome, Italy; ^2^Department of Surgical Sciences, Sapienza University of Rome Rome, Italy; ^3^Department of Medical, Technological and Translational Sciences, Ospedale di Cattinara, University of Trieste Trieste, Italy; ^4^Department of Public Health and Infectious Diseases, Sapienza University of Rome Rome, Italy; ^5^Department of Chemical Science and Technologies, University of Rome “Tor Vergata” Rome, Italy

**Keywords:** breast cancer, DHA, omega-3 index, omega-3 fatty acids, BRCA

## Abstract

**Rationale:** Docosahexaenoic acid (DHA) in cell membrane may influence breast cancer (BC) patients' prognosis, affecting tumor cells sensitivity to chemo- and radio-therapy and likely modulating inflammation. The possibility of identifying BC patients presenting with low DHA levels and/or low ability of DHA incorporation into cell membrane might help to treat this condition.

**Methods:** We enrolled BC patients and healthy controls, recording their seafood dietary intake. DHA in form of algal oil was administered for 10 consecutive days (2 g/day). Blood samples were collected at baseline (T0) and after 10 days of supplementation (T1) to assess DHA, omega-3 index, as the sum of DHA + eicosapentaenoic acid (EPA), in red blood cells (RBC) membranes and plasma tumor necrosis factor-alpha and interleukin-6 levels. Pre- and post-treatment fatty acid profiles were obtained by gas-chromatography. Parametric and non-parametric tests were performed, as appropriate, and *P*-value < 0.05 was considered statistically significant.

**Results:** Forty-three women were studied, divided into 4 groups: 11 patients with BRCA1/2 gene mutation (M group), 12 patients with familiar positive history for BC (F group), 10 patients with sporadic BC (S group), and 10 healthy controls (C group). DHA and omega-3 index increased from T0 to T1 in the 3 groups of BC patients and in controls (*P* < 0.001). No difference was found in DHA incorporation between each group of BC patients and between patients and controls, except for M group, which incorporated higher DHA levels with respect to controls (β = 0.42; *P* = 0.03). No association was documented between cytokines levels and DHA and omega-3 index at baseline and after DHA supplementation. Independent of the presence of BC, women considered as “good seafood consumers” showed at baseline DHA and omega-3 index higher with respect to “low seafood consumers” (*P* = 0.04; *P* = 0.007, respectively). After supplementation, the increase in DHA levels was greater in “low seafood consumers” with respect to “good seafood consumers” (*P* < 0.0001).

**Conclusion:** DHA supplementation was associated with increased DHA levels and omega-3 index in RBC membranes of BC cancer patients, independent of the type of BC presentation, and in controls. BRCA1/2 mutation, as well as low seafood consuming habits in both BC patients and healthy controls, seem to be associated with greater ability of DHA incorporation. Larger samples of BC patients are necessary to confirm our observation.

## Introduction

Breast cancer (BC) is the most common cancer in women, with an incidence greater than 1 million of new cases per year worldwide (Bougnoux et al., 2009, 2010). A strong relationship exists between diet, overweight and risk of primary BC and its recurrence (Amadou et al., 2013; Molfino et al., 2016). Diets with a high content in omega-6 polyunsaturated fatty acids (PUFAs), and relatively low omega-3 PUFAs (Molfino et al., 2014), are associated with increased risk to develop BC and its relapse (Chlebowski et al., 2006; de Lorgeril and Salen, 2012; Laviano et al., 2013). Moreover, omega-3 PUFAs, in particular the docosahexaenoic acid (DHA), are able to influence the efficacy of chemo- and radio- therapy in BC patients (Bougnoux et al., 2009, 2010), sensitizing the malignant tumor cells to chemo- and radio-therapy, not increasing the toxicity on non-tumor tissues (Bougnoux et al., 2010; Hajjaji et al., 2011; Laviano et al., 2012). The lipid environment of cancer cells (i.e., the lipid component of cell membranes) may influence tumor sensitivity to chemotherapeutic agents. The membrane lipids of cancer cells are similar to those of storage lipids in terms of fatty acids composition, suggesting that DHA levels may potentially influence the activity of anti-tumor agents (Bougnoux et al., 1999, 2010). The dietary supplementation with DHA might be able to increase the effectiveness of systemic chemotherapy and local mammary irradiation. In particular, DHA from food or from exogenous supplementation, after intestinal absorption, is rapidly incorporated into circulating phospholipids and in those of cell membranes, including the red blood cells (RBC). A study showed that in BC patients the incorporation of DHA in circulating phospholipids is variable, and two different phenotypes of BC patients were identified: “high-incorporator” (with high incorporation of DHA in phospholipids) and “low-incorporator” (with poor or reduced incorporation of DHA in phospholipids) (Bougnoux et al., 2009). The possibility of recognizing “low-incorporator” patients would identify patients who might significantly increase the intake of DHA to increase the sensitivity of the tumor to cancer therapies. In addition, it could be also hypothesized that “low-incorporators” may have an increased susceptibility to develop BC or a tumor recurrence.

Studies have been conducted on a possible relationship between PUFAs metabolism and the pathways involving the Breast Related Cancer Antigens (BRCA) 1 and BRCA2 genes (Bernard-Gallon et al., 2002), which are implicated in inherited predisposition to BC, showing the presence of a possible transcriptional or post-transcriptional regulation of BRCA1 and BRCA2 after omega-3 PUFAs treatment in breast tumor cells (Bernard-Gallon et al., 2002; Shiovitz and Korde, 2015).

Omega-3 fatty acids are incorporated into phospholipids of cell membranes during both reticulocyte maturation and through plasma exchange, making erythrocytes an accurate indicator of dietary fatty acid intake. The omega-3 index measures the percentage of the long-chained omega-3 fatty acids, eicosapentaenoic acid (EPA) and DHA, to total erythrocyte membranes fatty acids (Harris, 2008).

In this light, we aimed at assessing the ability of DHA incorporation in RBC membranes, expressed as omega-3 index, in BC patients and in healthy controls and the potential differences in the DHA incorporation ability, and at determining whether the incorporation of DHA could differ in BC patients with a family history of breast malignancy, either positive or not for BRCA1/BRCA2 gene mutation. We secondarily verified a possible association between omega-3 PUFAs levels in RBC membranes and the inflammatory status.

## Materials and methods

This was a spontaneous, single-center, controlled study performed on patients from the Department of Surgical Sciences, Sapienza—University of Rome, Italy. After approval of the local Ethics Committee and after obtaining written informed consent from each participant, women with diagnosis of BC and healthy women with no personal and no family history of BC were recruited. All procedures were in accordance with the ethical standards of the Helsinki Declaration issued in 1975 and later amendments. Exclusion criteria were: self-reported consumption of omega-3 PUFAs supplements and omega-3 PUFAs supplemented foods in the previous 6 months.

The sample size was determined based on a previous study conducted in BC patients observing changes of DHA levels before and after DHA oral supplementation (Bougnoux et al., 2009).

### Participants

We recorded participant's demographic and anthropometric characteristics (age, weight, height, body mass index—BMI) and serum nutritional and metabolic biomarkers, including cholesterol, low density lipoprotein (LDL), high-density lipoprotein (HDL), triglycerides. Histological diagnosis, tumor staging, and a detailed medical history were collected. Based on the familiar and past medical history, the participants were divided in: patients with no family history of breast malignancy—sporadic (S) group, patients with BC familial history, but negative for BRCA1 or BRCA2 gene mutation—familiar (F) group, patients with documented BRCA1 or BRCA2 gene mutation—mutated (M) group, healthy subjects—control (C) group matched for age and BMI. Participants were interviewed regarding the presence of body weight change over the prior 6 months, and for the presence of comorbidities such as diabetes, hypercholesterolemia, and hypertriglyceridemia. Questionnaire on participant's self-reported dietary habits was administered. It included questions on the habitual consumption of seafood in the diet, focusing on portion size (i.e., at least a seafood portion of 80 g) and frequency (once a month/once a week/more than once a week), as previously validated (Dahl et al., 2011). Based on the answers given, participants were divided into 2 groups: those who self-reported eating seafood in the diet once per month or less than once per month (“low seafood consumer”), and those who self-reported consuming seafood once per week or more than once per week (“good seafood consumer”).

### Intervention

DHA in the form of algal oil syrup (from Schizochytrium sp.) containing DHA at 10% (strawberry-flavored Richoil® syrup, DMF, Italy) was administered in patients and controls. The product was provided free of charge by the manufacturer. Each participant took 10 ml of the syrup twice per day for 10 consecutive days, corresponding to 2 g of DHA per day. A standard normo-balanced diet was prescribed during the same days, as well as to maintain the usual physical activity level. The participants were supplied with reference telephone number to contact for ensuring compliance and to discuss any difficulties during intervention period.

### Blood sample collection

Blood samples were collected at baseline (T0), and after DHA supplementation (after 10 days, T1). Whole blood samples were collected on overnight fasting by vein puncture in serum tubes and in ethylenediaminetetraacetic acid (EDTA) tubes, which were kept into ice and centrifuged at 3,000 rpm for 10 min at +4°C. After removing the plasma and, carefully, the buffy coat, the RBC aliquots were stored at −80°C and then analyzed.

### RBC fatty acid assay

RBC fatty acids composition was analyzed by gas chromatography—flame ionization detection (GC-FID; GC 6850 Agilent Technologies, Santa Clara, CA, USA), as previously described (Mazzucco et al., 2010), in the Laboratory of the University of Trieste, Italy. Laboratory personnel were unaware of the clinical status of the participants (i.e., BC patients or controls, type of intervention, dietary habits). Specific fatty acids standards were used to identify fatty acid methyl esters (FAME) by retention times in erythrocyte samples. Area-under-the-curve of each selected peak was determined by highly standardized hand integration performed using commercial software (HP Chem station; Agilent Technologies, Santa Clara, CA, USA).

RBC membrane level of each fatty acid was expressed as percent ratio between area-under-the-curve of each selected FAME peak and the sum of all measured FAME peaks.

Omega 3 index was calculated as sum of the DHA + EPA in erythrocyte membranes, indicating a percentage of total erythrocyte fatty acids.

### Serum cytokines analysis

Cytokines interleukin (IL)-6 and tumor necrosis factor (TNF)-alpha levels were measured in duplicate by commercially available ELISA kits (Abcam, Cambridge, U.K.) on the blood samples collected at T0 and T1.

### Statistical analyses

Patient's characteristics were described using mean ± standard deviation (SD) for continuous normally distributed variables, including DHA levels and omega-3 index overall and separately by group, and percent for dichotomous variables. Not-normally distributed variables were described using median (25th, 75th percentiles). Interactions between treatment and participant characteristics (age, BMI, body weight change) were tested to identify inter-individual differences in omega-3 index response to treatment.

One-sample *t*-tests were used to test for overall change from T0 to T1. Pearson's correlation was used to analyze the association between inflammatory markers and DHA and omega-3 index, at baseline and after supplementation. Categorical variables were utilized using proportions by χ^2^ test. Multivariable regression analysis, adjusting for patient characteristics (i.e., age, BMI, comorbidity, inflammation, BC presentation, seafood consumption), was performed to predict DHA and omega-3 index changes before and after supplementation. Adjusted *P* < 0.05 was considered significant. All statistical analyses were performed in R v. 3.0.2.

## Results

### Participant's characteristics

A total of 45 women were enrolled, including patients and controls. Two subjects withdrew from the study between T0 and T1, because no longer interested in the study (1 patient in M group and 1 healthy control). Forty-three participants, 33 BC patients and 10 healthy women (C group) completed the study. Breast cancer patients were distributed as follows: 10 patients in S group, 12 patients in F group, 11 patients in M group. Baseline characteristics of the participants are shown in Table 1. Mean age was 47.3 ± 8.9 years for BC patients and 48.3 ± 5.66 years for group C.

**Table 1:** Participants' characteristics.

**All participants *N* = 43**	BC patients *N* = 33, Mean ± SD^*^	Controls *N* = 10, Mean ± SD^*^
Age, years	47.33 ± 8.88	48.3 ± 5.66
Body weight, kg	63.91 ± 11.84	60.6 ± 7.4
BMI, weight (kg)/height^2^ (m)	23.93 ± 3.98	23.68 ± 2.93
Glycemia, mg/dl	94.1 ± 9.57	88.72 ± 9.93
Cholesterol, mg/dl	206.8 ± 28.63	217.7 ± 31.77
**Comorbidities:**		
Diabetes mellitus (yes/no)	2/31	0/10
Hyperlipidemia (yes/no)	7/26	1/10
IL-6, pg/ml	2.12 (0.9, 9.08)	4.04 (0.78, 7.85)
TNF-alpha, pg/ml	26.04 (12.32, 116.37)	15.3 (9.61, 140.15)
Seafood consuming (yes/no)	25/8	8/2

* *Median (interquartile range) is shown for non-normally distributed variable (IL-6, TNF-alpha)*.

*BC, breast cancer; BMI, body mass index; IL, interleukin; TNF, tumor necrosis factor*.

### DHA levels and omega-3 index in RBC membranes at baseline and after supplementation and the role of inflammation

At baseline, no significant differences were observed in DHA levels and omega-3 index between BC patients and controls, neither between each group of BC patients (S, F, M group).

All the participants took the DHA oral supplementation. During and after supplementation, the daily doses of oral DHA were well tolerated with good compliance in all the participants.

After supplementation, DHA levels and omega-3 index significantly increased in all groups of BC patients and in the controls (*P* < 0.001) (Table 2). No differences in DHA incorporation and omega-3 index were observed between the three groups of BC patients and between patients and controls, except for M group showing higher ability of DHA incorporation when compared to healthy women (C group) (β = 0.30; *P* = 0.02) (Table 3).

**Table 2:** DHA levels, EPA levels, and omega-3 index in RBC membranes in the four groups of participants at baseline (T0) and after DHA supplementation (T1).

**All participants (*N* = 43)**	**T0, Mean ± SD**	**T1, Mean ± SD**	***P***
**S GROUP (*N* = 10)**			
DHA	6.14 ± 1.21	7.54 ± 0.97	0.002
EPA	0.53 ± 0.23	0.68 ± 0.22	0.014
Omega-3 Index	6.67 ± 1.33	8.21 ± 1.08	0.002
**F GROUP (*N* = 12)**			
DHA	6.09 ± 1.11	7.35 ± 1.12	0.003
EPA	0.62 ± 0.27	0.77 ± 0.33	0.004
Omega-3 Index	6.71 ± 1.32	8.12 ± 1.35	<0.001
**M GROUP (*N* = 11)**			
DHA	5.86 ± 1.51	7.4 ± 1.32	<0.001
EPA	0.60 ± 0.34	0.7 ± 0.27	0.018
Omega-3 Index	6.43 ± 1.75	8.1 ± 1.53	<0.001
**C GROUP (*N* = 10)**			
DHA	6.1 ± 0.88	7.23 ± 0.7	0.006
EPA	0.52 ± 0.11	0.6 ± 0.1	0.004
Omega-3 Index	6.62 ± 0.9	7.0 ± 0.74	0.002

*DHA, docosahexaenoic acid; EPA, eicosapentaenoic acid; RBC, red blood cell; S group, sporadic group; F group, familiar group; M group, mutated group; C group, controls*.

**Table 3:** Multivariate regression models to predict variation of DHA levels in RBC membranes between baseline (T0) and after DHA supplementation (T1).

**Clinical characteristics**	**Beta coefficient (95% CI)**	***P***
**TYPE OF BC PRESENTATION**		
S group vs. C group	0.12 (−0.21, 0.44)	0.47
F group vs. C group	0.21 (−0.13, 0.55)	0.21
M group vs. C group	0.30 (0.05, 0.55)	0.02
**DIETARY SEAFOOD HABITS**		
Good vs. Low seafood consumer	−0.49 (−0.68, −0.30)	<0.001

*DHA, docosahexaenoic acid; RBC, red blood cell; BC, breast cancer; S group, sporadic group; F group, familiar group; M group, mutated group; C group, controls*.

At baseline, no differences in TNF-alpha and in IL-6 were documented in the four groups (Table 1), and no correlation was found between DHA levels or omega-3 index and cytokines levels at baseline and after supplementation.

### Self-reported dietary seafood consumption, DHA, and omega-3 index

According to the self-reported seafood eating habits, 33 participants resulted as “good seafood consumers,” 25 BC patients (7 in S, 11 in F, and 7 in M group) and 8 controls. Ten participants, 8 BC patients (3 in S, 1 in F, and 4 in M group) and 2 controls resulted as “low seafood consumers” (Tables 1, 4). At baseline “good seafood consumers” showed higher DHA levels and omega-3 index with respect to “low seafood consumers” (Table 4) (*P* = 0.01). After supplementation, we observed a significant increase in DHA levels and omega-3 index in “good seafood consumers” (*P* < 0.001) (Table 4), as well as in “low seafood consumers” (*P* = 0.002 and *P* = 0.006, respectively) (Table 4). The increase in DHA levels was higher in “low seafood consumers” with respect to “good seafood consumer” (*P* < 0.001) (Table 3). No association was found between the type of BC presentation and seafood consumption.

**Table 4:** DHA levels, EPA levels and omega-3 index in RBC membranes in “good” and “low seafood consumers” at baseline (T0) and after DHA supplementation (T1).

**All participants (*N* = 43)**	**T0, Mean ± SD**	**T1, Mean ± SD**	***P***
**GOOD SEAFOOD CONSUMERS (*N* = 33)**			
DHA	6.29 ± 1.10	7.5 ± 1.03	<0.001
EPA	0.61 ± 0.29	0.74 ± 0.26	<0.001
Omega-3 Index	6.9 ± 1.26	8.24 ± 1.19	<0.001
**LOW SEAFOOD CONSUMERS (*N* = 10)**			
DHA	5.24 ± 1.04	6.97 ± 0.97	0.002
EPA	0.41 ± 0.09	0.52 ± 0.12	0.014
Omega-3 Index	5.64 ± 1.09	7.49 ± 1.05	0.006

*DHA, docosahexaenoic acid; EPA, eicosapentaenoic acid; RBC, red blood cell*.

## Discussion

Several studies have addressed the therapeutic effects of omega-3 PUFAs in cancer showing that omega-3 PUFAs can improve efficacy and tolerability of chemotherapy (Bougnoux et al., 2009; Nabavi et al., 2015). There are clinical trials where DHA alone or combinations of omega-3 PUFAs are being tested for cancer prevention, support, or therapy (Berquin et al., 2008; Nabavi et al., 2015). DHA as a treatment strategy is often combined with chemotherapeutic drugs since DHA most likely enhances the cytotoxic effects of these drugs (Nabavi et al., 2015).

Our study aimed at verifying the ability of DHA incorporation in RBC membranes of BC patients after oral DHA supplementation compared to heathy women. We found that DHA levels, and omega-3 index had a significant increase after a short period of supplementation (10 days) in BC patients, with no difference related to the type of BC presentation, as well as between patients and controls. Interestingly, we observed that only M group had significantly higher DHA increase with respect to control group. Arterburn et al. (2006) found that DHA supplementation in healthy humans led to a dose-dependent increase in RBC DHA and plasma phospholipid and contents. The data available in the literature are mostly centered on the effect of DHA supplementation during chemo- and radio-therapy in reducing adverse side effects and in improving the outcome of chemotherapy, when highly incorporated (Bougnoux et al., 2009). Also, studies in experimental models showed that, under DHA-supplemented diet, peroxisome proliferator-activated receptors β (PPARβ) is a crucial player capable of regulating different PPAR mRNA expressions, which downregulate BC cell growth and mammary tumor growth (Wannous et al., 2013). The possibility of recognizing in BC patients potential differences in DHA absorption was previously evaluated (Bougnoux et al., 2009). In fact, in a phase II trial, metastatic BC patients were categorized in “low or high incorporator” according to patient's ability to incorporate oral DHA supplementation (Bougnoux et al., 2009). Interestingly, authors documented that DHA “low incorporators” showed a worse outcome in term of reduced response to therapy and increased side effects, such as anemia and thrombopaenia (Bougnoux et al., 2009). In this respect, we were not able to demonstrate in our study a similar behavior in BC patients. In particular, we did not identify one or more groups of patients with this characteristic, possibly because of several factors, including age (Walker et al., 2014), that might have influenced the difference in DHA incorporation described in our study and by Bougnoux et al. (2009).

Studies in BC patients, where DHA was combined with the chemotherapeutic drugs epirubicine, cyclophosphamide, and 5-fluorouracil, emphasize that an interindividual uptake, and incorporation of DHA can alter the treatment response (Bougnoux et al., 2009) and reduce BC cell proliferation (Corsetto et al., 2012). Patients were supplemented with DHA daily during the chemotherapy cycles and were then divided into high and low incorporating groups based on the DHA levels in plasma and RBCs. The high incorporating group was characterized by delayed time to tumor progression and longer overall survival compared to the low incorporating group (Bougnoux et al., 2009). This observation is in line with other studies showing that DHA incorporation differs between individuals due to dissimilar rates of metabolism, enzymatic activity, background diet, age, and gender (Arterburn et al., 2006; Rusca et al., 2009).

The clinical setting in which the patients were previously treated for a longer duration of time is different from ours. In fact, BC patients were supplemented during the entire duration of chemotherapy (Bougnoux et al., 2009). Our patients were not on chemotherapy and were orally supplemented with DHA to assess the capacity of incorporation and not to observe effect associated with anticancer treatments.

Moreover, when we considered in all the participants the dietary seafood habits, which were considered a reliable instrument to assess omega-3 fatty acids intake (Dahl et al., 2011), we found that “good seafood consumers” had higher DHA levels at baseline with respect to “low seafood consumers,” confirming the reliability of our questionnaire.

After supplementation “low seafood consumers” showed higher DHA increase with respect to “good seafood consumers.” One possible explanation is represented by the fact that “good seafood consumers” at baseline presented with higher DHA levels, and possibly, reaching the maximum rate of absorption of DHA. In this light, our data are in accordance with those obtained in the study by Bougnoux et al. (2009), where the highest DHA levels described in “high incorporators” after supplementation reached values comparable to the ones obtained in our study.

Since DHA is a highly unsaturated PUFA, it is susceptible to peroxidation and can cause accumulation of a surplus of reactive oxygen species that cannot be scavenged by the cancer cells. Addition of anti-oxidants to cells incubated with DHA diminishes the toxic effects, strengthening this theory (Lindskog et al., 2006; Gleissman et al., 2010). In our study we did not assess this specific effect.

Several metabolic derangements are described in BC patients, mostly represented by insulin-resistance and alterations in lipid metabolism (Amadou et al., 2013; Molfino et al., 2016). However, in our cohort of BC patients we did not observe a high prevalence of diabetes (only 2 patients), and this might have probably reduced the possibility to observe effect of DHA supplementation in this clinical setting.

In the recent years, the composition of the cell membrane fatty acids has been investigated, not only as a factor influencing the response to treatments, but also as an element influencing BC prognosis independent of the treatment received (Bougnoux et al., 2009, 2010; Straka et al., 2015). Therefore, the identification of different cell membrane composition at baseline, and a possible variation in DHA incorporation among the different type of BC presentation, could be useful in clinical setting. In this light, our data reveal differences only between BRCA mutation carriers (M group) and controls. In fact, although patients of M group did not show differences at baseline in terms of DHA levels and omega-3 index with respect to the other groups, they showed greater and significant increase of DHA levels and omega-3 index after supplementation when compared to healthy women. We are not able to describe a mechanism underlying this behavior and we cannot exclude the possibility that reduced basal DHA level in BRCA mutation carriers might be at least in part determined by low dietary DHA intake and/or by impaired absorption of food-derived DHA amount.

BRCA is the major tumor suppressor gene associated with hereditary predisposition to BC, and the risk of BC is known to be increased by a lack of BRCA1/2 protein function (Shiovitz and Korde, 2015). Interestingly enough, an experimental study found that DHA supplementation significantly reduced the incidence of BC and led to 60% increase in BRCA1 protein level with respect to the control group (not supplemented with DHA), indicating that BRCA1 up-regulation mediated by DHA might be protective against the risk to develop BC (Jourdan et al., 2007). Moreover, Bernard-Gallon et al. observed an increase of BRCA1 and BRCA2 mRNA expressions in DHA-treated BC cell lines, suggesting the presence of a possible transcriptional or post-transcriptional regulation of BRCA1 and BRCA2 genes after omega-3 PUFAs treatment in BC cells (Bernard-Gallon et al., 2002).

Linking BRCA to key nutritional factors, such as omega-3 PUFAs, involved in the incidence rate of BC (Brasky et al., 2010; Molfino et al., 2016), opens wide perspectives for nutritional prevention in BC and in possibly modulating inflammatory status. Recently, Roy et al. documented positive associations between erythrocyte and breast tissue omega-3 fatty acids, and suggestive inverse associations between erythrocyte long chain omega-3 PUFAs and tissue C-reactive protein (CRP) (Roy et al., 2015).

Although inflammation is a common condition in BC, our data fail to reveal increased circulating levels of pro-inflammatory cytokines in BC patients and no modification of IL-6 and TNF-alpha were obtained after DHA supplementation. This is in line with recent data available in the literature, demonstrating that inflammation is more common in obese BC patients (Gershuni et al., 2017), although we did not asses high-sensitivity CRP, which might give additional important information. In fact, our cohort showed a normal average BMI and it did not differ between BC patients and controls. Moreover, a recent randomized trial, conducted in healthy young adults daily supplemented with oral EPA + DHA for 5 months, showed a marginal decrease in serum TNF-alpha and no change in IL-6 levels (Flock et al., 2014).

Although our study involved a homogeneous population of BC patients, enrolled in a single cancer unit, all receiving the same DHA supplementation and the intervention being conducted only by one medical team, it presents several limitations. Our cohort of BC patients is small and may not be representative of larger BC patients' population. The small sample size of the groups enrolled might have limited the possibility of identifying association between patient's characteristics, in particular inflammatory status, and basal DHA and omega-3 index and DHA absorption overtime (during and after supplementation). Additionally, the dietary assessment tool utilized to assess seafood intakes, as an estimate of omega-3 PUFAs intake, might not be accurate, because it does not include questions on omega-3 PUFAs intakes from other dietary sources.

In conclusion, our study serves as the basis for development of larger trial to clarify the clinical impact of different DHA levels and omega-3 index in BC and, in particular, if DHA supplementation, performed for a short, as we did, or longer duration of time, has an impact on robust outcomes and if different subgroups of patients (i.e., BRCA mutation carriers) present a different and clinically relevant behavior and to potentially develop novel therapeutic strategies.

## Ethics statement

The local Ethics Committee (Policlinico Umberto I, Sapienza University of Rome, Italy) approved the study. Consent procedure for human research was obtained. All procedures were in accordance with the ethical standards of the Helsinki Declaration issued in 1975 and later amendments.

## Author contributions

AM designed research, conducted research, analyzed data, wrote the paper. MA conducted research, analyzed data, wrote the paper. SM and CR performed laboratory dosages. GB provided essential reagents necessary for research and analyzed and interpreted laboratory data. AF performed statistical analysis. SA interpreted laboratory data. MMo reviewed the paper. MMu designed research, reviewed the paper, and had primary responsibility for final content. All authors read and approved the final manuscript.

### Conflict of interest statement

The authors declare that the research was conducted in the absence of any commercial or financial relationships that could be construed as a potential conflict of interest.
